# Measuring Dynamic Gradients in Drying Battery Electrode Coatings via Microscale Resistivity

**DOI:** 10.1021/acs.langmuir.5c04644

**Published:** 2025-10-28

**Authors:** Emre Baburoglu, Karla Negrete, Maureen H. Tang, Nicolas J. Alvarez

**Affiliations:** † Materials Science and Engineering, 6527 Drexel University, Philadelphia, Pennsylvania 19104, United States; ‡ Mechanical Engineering and Mechanics, Drexel University, Philadelphia, Pennsylvania 19104, United States; § Chemical and Biological Engineering, Drexel University, Philadelphia, Pennsylvania 19104, United States

## Abstract

In situ techniques for probing the microstructural evolution of lithium-ion battery (LIB) electrodes are often limited by the cost or accessibility. This study demonstrates the use of a simple and cost-effective four-line probe device to measure dynamic electrode microstructures at varying penetration depths and to explain the effects of shear during coating on the transient and final electrode microstructure. Previous studies report superior performance for LIB electrodes coated at a high shear rate over those coated at a low shear rate. The researchers postulated that this was due to a difference in carbon connectivity in the final dried electrode, and this difference was partially supported by energy dispersive spectroscopy (EDS) atomic distribution analysis. In this study, we revisit these coating conditions at high and low shears to determine the time evolution effect of shear on the carbon microstructure formed during drying. The electrode resistances at different penetration depths clearly show a difference in the dynamic microstructure for different shear rates, indicating different drying mechanisms. Heuristic drying models are used to interpret resistivity data of the two electrodes and propose the drying mechanisms. For example, at low shear rates, there is obvious aggregation and sedimentation of carbon particles at early times. Furthermore, we observed the formation of a carbon-rich top layer during drying for both shear rates. Electrochemical fluorescence microscopy (EFM) and EDS imaging of the final dried electrode validate the observations determined from the resistivity measurements and modeling. Overall, these results use a low-cost, in situ method to offer a comprehensive understanding of how the shear rate influences the microstructural development of composite electrodes during drying and its implications for battery performance.

## Graphical abstract

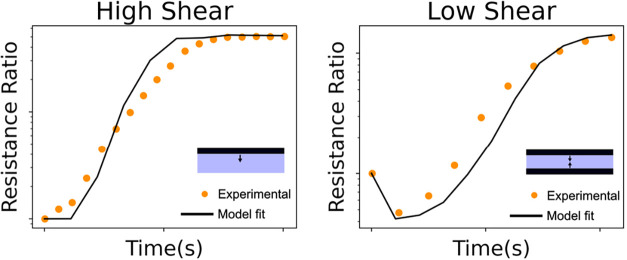

## Introduction

The performance of a composite lithium-ion battery electrode is largely dependent on its final carbon distribution.^1–5^ Despite this, there is no universally accepted method of obtaining or even identifying the ideal carbon distribution during electrode processing. Contrasting literature results suggest that the ideal carbon distribution depends strongly on the formulation of the battery electrode in question. For example, many studies have found that a homogeneous distribution of carbon leads to the best performance.^5–7^ However, in a study in 2020, Yari and co-workers found that a higher concentration of carbon black near the current collector improved the electrode performance.^8^ Xu and Gao found similar results for a SiO/graphite composite anode through their multiphysics modeling simulations.^9^ Some groups, including our own, claim that the distribution of carbon with respect to the active material particles is as important as the distribution of carbon with respect to the current collector.^1,2^ This is especially true for cathodes containing highly resistive active materials such as lithium iron phosphate (LFP).^10^ To address this gap in knowledge, it is necessary to understand the impact of electrode processing parameters resulting in the final carbon distribution for each electrode formulation.

In a previous study from our group, we showed that shear rate (γ̇) and drying temperature impact the final carbon distribution in the electrode significantly, which impacts the performance.^1^ Although we were unable to determine the drying mechanisms that led to different carbon distributions, we were able to measure the final distribution of carbon through EDS imaging of dried electrodes. This type of ex situ analysis of dried electrodes is the standard technique in the literature of studying film microstructure^11–15^ because methods to investigate microstructure evolution in situ during drying are expensive, laborious, and can only characterize part of the film. For example, Jaiser et al. froze electrode films at different time points in the drying process and imaged the planar cross section via scanning electron microscopy (SEM).^16^ Although this time-consuming process generates a clear representation of discrete time points in the drying process, it assumes reproducibility of the drying mechanism over many different films and requires the use of expensive equipment. Another example was the use of fluorescence microscopy by Lim et al. to determine the development of vertical particle distribution in an LIB anode as it dried but relied upon the introduction of fluorescent latex particles whose effect could not be deconvoluted.^17^ In a low-cost technique, Kumberg et al. used digital microscopy to image an LIB anode film coated on a glass slide from beneath. However, this method proved insufficient to characterize the drying mechanism.^18^

Recently, a simple and cost-effective electrical resistivity method was proposed to detect microstructural changes in a drying film.^19^ Resistivity is measured using two sets of four-line probes: one with electrode spacing (*P*) much larger than the initial film thickness (*H*_0_), and another with *P* significantly smaller than *H*_0_. The larger-spaced probe measures the resistivity of the entire film, while the smaller-spaced probe measures only the resistivity at the bottom of the film. This enables the detection of changes in resistivity as a function of film thickness and time, which can then be linked to the drying mechanism via a heuristic drying model analysis. Since in the case of LIB electrodes, the carbon particles are the only electrically conductive component, this method is able to monitor the carbon distribution and connectivity as the film dries. In this work, we use this method to study the impact of the coating shear rate on the evolution of the carbon microstructure of an LIB cathode during drying. The dynamic microstructural analysis is related to the final electrode microstructure measured using SEM image analysis and electrochemical fluorescence microscopy (EFM)^20^ of the final dried film. Overall, this technique proves to be very capable of measuring drying dynamics and understanding the effect of processing conditions on the evolution of the electrode microstructure.

## Materials and Experimental Procedures

### Electrode Film Composition and Mixing

Electrode films were made with 95 wt % active material of LiNi_0.33_Mn_0.33_Co_0.33_O_2_ (NMC) (MTI Corporation) with an average particle size of 10 μm and a particle density of 4.55 g/cm^2^. The conductive additive consisted of 2.5 wt % carbon black (CB) (MTI, Super C65) with a particle density of 2 g/cm^2^. The active material and conductive additive were added to a solution of 4.6 wt % poly­(vinylidene difluoride) (PVDF) (Arkema, MW = 380k) in 3.405 mL of 1-methyl-2-pyrrolidone (NMP, Sigma-Aldrich), which was mixed separately in a Thinky planetary mixer at 1800 rpm for 10 min. The PVDF solution and particles were combined and mixed in the same device for 10 min at 1800 rpm. The final dried electrode composition remained constant through all trials at 95% active material, 2.5% CB, and 2.5% PVDF binder.

### Resistance Measurements

Microscopic probes for resistance measurements were manufactured by using standard photolithography procedures in the Singh Center for Nanotechnology. *P* values were selected as 1 mm for the larger-spaced probes and 10 μm for the smaller-spaced probes. More details on the fabrication procedures and selection of probe spacings can be found in our previous publication.^19^ All electrodes were 25 mm in length, as shown in Figure 1.

**Figure 1:**
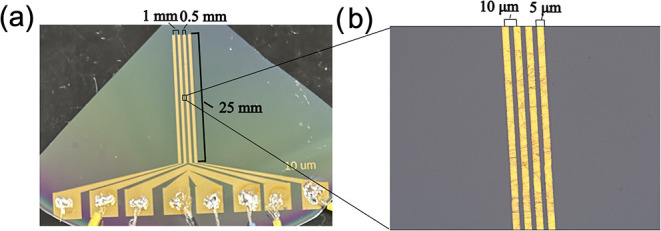
(a) Image of fabricated four-line probe device with center-to-center probe spacing and electrode width of the larger-spaced probes labeled. (b) 50× magnified image of the smaller-spaced probes in the middle of the larger-spaced probes with center-to-center probe spacing and electrode width labeled.

After fabrication, the electrode slurry was coated on the line probes via an automatic coater by using a doctor blade at a wet thickness of 100 μm. This thickness was validated using a Keyence VR-6000 profilometer. Films were coated at blade speeds of 30 and 130 mm/s. Blade speeds were measured using video analysis. The corresponding shear rates are γ̇ = 300 and 1300 s^–1^, respectively. All films were air-dried at room temperature under a snorkel. Each dynamic resistance experiment was replicated three times. We attribute variations in drying times to uncontrolled variation in environmental factors such as humidity, exact room temperature, and air currents in the lab. During drying, a VMP-3 potentiostat (Biologic USA) was used to monitor the resistance using two different electrochemical techniques, depending on the probe spacing. For the larger-spaced electrodes, potentiostatic electrochemical impedance spectroscopy (PEIS) was performed at 20 μV and 10 Hz. For the smaller-spaced electrodes, resistance was measured with cyclic voltammetry (CV) using a scan rate of 20 mV/s over a voltage range of −0.01 to 0.01 V. Different measurement techniques were selected based on experimental observations that each method minimized data noise for its respective probe configuration. The underlying reason for this difference in the noise behavior remains unclear. The experimental setup is depicted in Figure 2(a).

**Figure 2:**
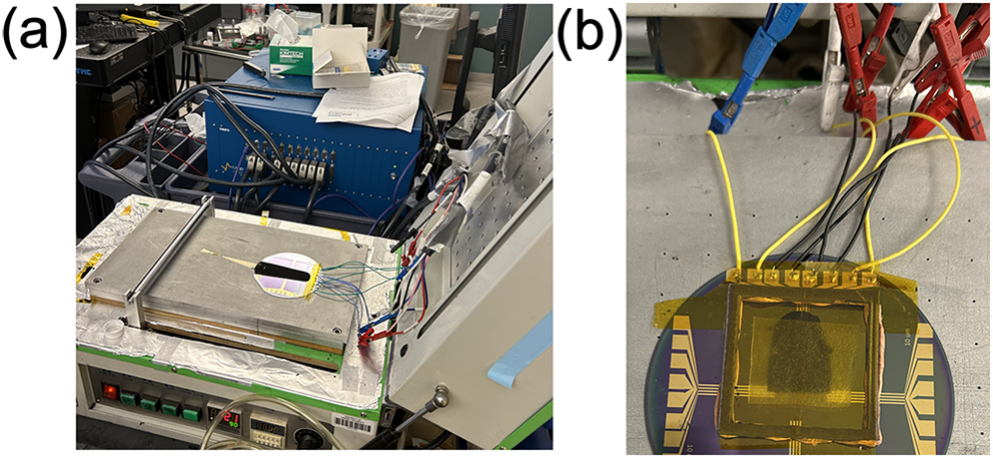
(a) Picture depicting the experimental setup with the VMP-3 potentiostat (blue) connected to the silicon wafer containing the device. (b) Experimental setup where the coating is covered to prevent drying.

#### No-Drying Measurements

Experiments in which drying was prevented were conducted by covering the film with a 5 cm × 5 cm polyoxymethylene (Delrin) frame, which was covered with Kapton tape and had a tissue soaked in NMP adhered to the tape. Silly Putty was used as an adhesive between the frame and the device to ensure an airtight seal. This experimental setup is shown in Figure 2(b).

### Characterization

Electrode slurries of identical formulations were coated on an aluminum current collector under the same conditions as described above. The films were air-dried in a fumehood and analyzed using methods described below.

#### Electrochemical Fluorescence Microscopy

EFM relies on the principle of electrofluorochromism, in which fluorescence is reversibly (de)­activated upon electrochemical redox. Here, particles of NMC that are well-connected to the carbon network reduce a fluorophore, 9,10-anthraquinone (AQ), while particles or regions with high electronic resistance do not. EFM was thus employed to visualize and quantify local electronic contacts between active material particles and the carbon network on the electrode surface.^20^ This analysis aimed to compare the concentration or connectivity of carbon black near the surface in films coated at 1300 s^–1^ versus those coated at 300 s^–1^. 10 mm electrode discs were imaged in an ECC-Opto-10 optical cell using a Zeiss AxioObserver wide-field microscope equipped while applying a constant current of 7.6 mAh/cm^2^ with a BioLogic potentiostat. Electronically disconnected pixels were quantified using a simplified image processing method of Negrete et al.^20^ Briefly, the pixel intensities were normalized and binarized by using a 65% median threshold. The percentage of the disconnected area was calculated by comparing the pixel counts above and below the threshold.

#### Scanning Electron Microscopy

SEM images (Apreo 2S) and energy dispersive spectroscopy (EDS) maps (EDAX Team) were measured (10 kV) on the same films used for EFM. The SEM and EDS images were collected in the same region of interest and analyzed to determine component distributions. The samples were prepared by vertically slicing electrodes into 12 mm × 5 mm rectangles with a razor blade. The electrode was then mounted to a 90° angle SEM stub with a conductive carbon tape, exposing the cross section. SEM/EDS images were cropped to include only the electrode cross section. The vertical carbon distribution in the films was quantified by integrating rows of the obtained EDS images to count the number of red pixels (colors assigned to carbon). The number of pixels was averaged over 6 images and plotted against the normalized thickness of the electrode to give the vertical carbon distribution.

#### Thickness Measurements

Thickness was measured with a microepsilon confocal DT IFC-2421. Figure 3 shows the measured decrease in the film height as the battery electrode coating dries at room temperature. The black line shows the exponential decay function used to approximate this change in film height in eq 2.

**Figure 3:**
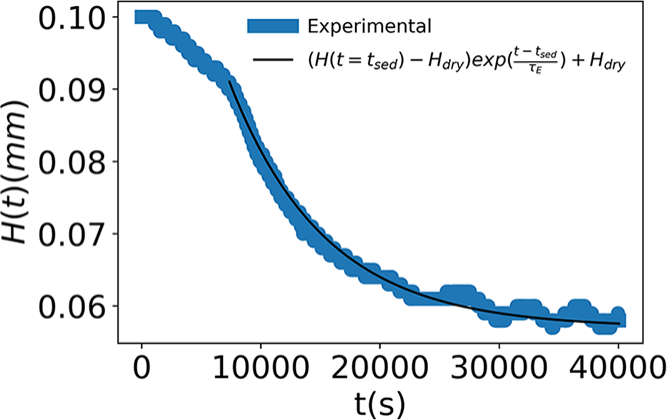
Measured change in thickness of a battery electrode coating as it dries at room temperature (blue) along with the model fit (black).

## Drying Model Development

A model simulating four-electrode resistance measurements during thin film drying was developed previously.^19^ The model solves Laplace’s equation for electric potential, ∇^2^ϕ = 0, in stacked layers. The top and bottom boundaries are treated as insulating, with a current *I* applied at one outer electrode and the other grounded. The potential ϕ is evaluated at the inner electrodes and the measured resistance is given by *R* = Δϕ/*I*. The height of the film is a function of time. The normalized initial film thickness was defined as *H*_0_/*P*_S_ = 10 and *H*_0_/*P*_L_ = 0.5, where *P*_S_ and *P*_L_ are the equidistant electrode spacings for the smaller- and larger-spaced probes, respectively. Note that *H*_0_/*P*_L_ = 0.5 satisfies the necessary condition to measure the resistance across the entire film, as detailed in ref 19. The corresponding measured resistances are denoted as *R*_S_ (small spacing) and *R*_L_ (large spacing), with calculated resistivities ρ_S_ and ρ_L_. The ratios of resistance and resistivity are given by α = *R*_S_/*R*_L_ and χ = ρ_S_/ρ_L_, which eliminates the effect of homogeneous drying and enables a clearer analysis of vertical gradients in concentration.

During drying, it is possible for distinct layers of concentrated regions to form due to sedimentation and fast evaporation. If the particles sediment faster than the film dries, a bottom layer of sediment forms, and the top layer is depleted of particles. If the film dries faster than the particles are able to move away from the air–liquid interface, a concentrated top layer is formed with a homogeneous bottom layer. If the particles diffuse faster than sedimentation or drying, the film dries homogeneously and there is only one layer, which is denoted by χ = 1 and α as a linear line with time. For the purpose of this work, we introduce a fourth case, described and characterized by Cardinal et al.,^21^ which involves simultaneous sedimentation and surface accumulation, resulting in a transient depleted middle layer. Figure 4 illustrates a vertical cross section of the geometry used in this model.

**Figure 4:**
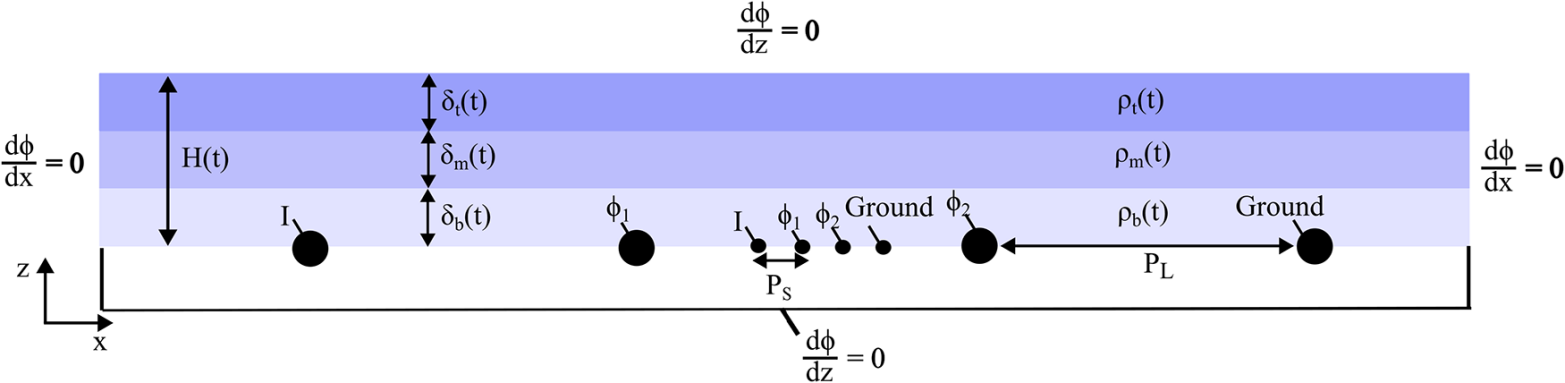
Schematic of the four-electrode measurement simulation on a three-layered film modeling a drying particulate film, including drying parameters and boundary conditions.

We assume that the film resistivity is a function of space and time, ρ­(*z*, *t*), depending on the local particle concentration, given by

1 $$\rho \left(z,t\right)=\{\begin{array}{l} \rho_{b}\left(t\right),if\;z\leq \delta_{b}\left(t\right)\\ \rho_{m}\left(t\right),if\;H\left(t\right)-\delta_{t}\left(t\right)>z>\delta_{b}\left(t\right)\\ \rho_{t}\left(t\right),if\;H\left(t\right)>z\geq H-\delta_{t}\left(t\right) \end{array}$$

where *z* is the vertical coordinate, *H*(*t*) is the total film thickness, δ_b_(*t*) is the bottom layer thickness, δ_m_(*t*) is the middle layer thickness, and δ_t_(*t*) is the top layer thickness. Note that not all layers are present in every case. The transient resistivities of the bottom, middle, and top layers are represented by ρ_b_(*t*), ρ_m_(*t*), and ρ_t_(*t*), respectively.

As shown in Figure 3, the film thickness during drying decreases slowly and linearly at short time *t* < 7000 s (less than 8 μm) and exponentially at long time, *t* > 7000 s. To simplify the model and reduce the number of fitting parameters, we assumed a constant thickness at a short time, when only sedimentation takes place. At long time, when sedimentation completes and the film evaporates, the change in thickness is approximated using an exponential decay function, given by

2 $$\frac{H\left(t\right)}{H_{0}}=\{\begin{array}{l} 1,if\;t\leq t_{sed}\\ \left(H_{0}-H_{dry}\right)\exp\left(-\frac{t-t_{sed}}{\tau_{E}}\right)+H_{dry},if\;t>t_{sed} \end{array}$$

where *H*_0_ is the initial thickness of the film, *H*_dry_ is the dry thickness, and τ_E_ is the evaporation time constant with τ_E_ = *t*_f_/5 where *t*_f_ is the time taken to reach constant resistivity in the film. *t*_f_ is taken from the experiment to be 40,000 s, and τ_E_ is fit to the exponential region of the experimental curve. *t*_sed_ is the sedimentation time, and the magnitude of *t*_sed_ is determined by the experimentally observed inflection point, e.g., 7000 s in Figure 3.

At short time, *t* < *t*_sed_, only sedimentation occurs. Sedimentation is modeled as a layer of constant resistivity, ρ_b_(*t*) = ρ_sed_, with a thickness δ_b_(*t*) = that grows linearly from the bottom up

3 $$\delta_{b}\left(t\right)=\{\begin{array}{l} \delta_{b}\frac{t}{t_{\mathrm{sed}}},\mathrm{if}\;t\leq t_{\mathrm{sed}}\\ \delta_{b},\mathrm{if}\;t>t_{\mathrm{sed}} \end{array}$$

where δ_b_ is the final layer thickness of the bottom layer (sediment).

For *t* > *t*_sed_, there can be either homogeneous drying of a given layer or a buildup of particles in the top layer. For homogeneous drying, the *i*th layer resistivity is given by

4 $$\rho_{i}\left(t\right)=\{\begin{array}{l} \rho_{0},\mathrm{if}\;t\leq t_{\mathrm{sed}}\\ \left(\rho_{0}-\rho_{i}\right)\exp\left(-\frac{t-t_{\mathrm{sed}}}{\tau_{E}}\right)+\rho_{i},\mathrm{if}\;t>t_{\mathrm{sed}} \end{array}$$

where ρ_0_ is the initial resistivity of the film, which is taken to be homogeneous at *t* = 0 and ρ_*i*_ is the final resistivity of layer *i*. For accumulation of particles in the top layer, the top layer thickness and resistivity is given by

5 $$\delta_{t}\left(t\right)=\{\begin{array}{l} 0,\mathrm{if}\;t\leq t_{\mathrm{sed}}\\ {\begin{array}{l} \delta \end{array}}_{t}\left(1-\exp\left(-\frac{t-t_{\mathrm{sed}}}{\tau_{E}}\right)\right),\mathrm{if}\;t>t_{\mathrm{sed}} \end{array}$$

6 $$\rho_{t}\left(t\right)=\{\begin{array}{l} \rho_{0},\mathrm{if}\;t\leq t_{\mathrm{sed}}\\ \left(\rho_{0}-\rho_{t}\right)\exp\left(-A\frac{t-t_{\mathrm{sed}}}{t_{f}}\right)+\rho_{\mathrm{skin}},\mathrm{if}\;t>t_{\mathrm{sed}} \end{array}$$

where δ_t_ is the final top layer thickness, ρ_t_ is the final resistivity of the top layer, and *A* is an estimated parameter determined from a best visual fit to the α­(*t*) experimental data. Unlike the sedimentation layer, the top layer concentration increases with time due to evaporation, and thus the resistivity is a function of time.

The resistivity of the four-point probe was simulated numerically in COMSOL Multiphysics 5.6 using the Electric Currents physics package with the stationary solver.

## Results and Discussion

Figure 5(a) shows the experimental change in *R*_L_ (filled circles) and *R*_S_ (unfilled circles) as a function of time as the electrode slurry coated under γ̇ = 1300 s^–1^ dries. As the film dried, the resistance measured by both probes decreases. This trend is attributed to the increasing concentration of conductive carbon black particles as the solvent evaporates, increasing the electrical connectivity. The decrease in *R*_S_ is significantly less than that of *R*_L_, indicating a difference in resistivity across the film thickness. This is better seen in Figure 5(b) with the corresponding α, where α is a strong increasing function of time, suggesting a higher resistivity of the bottom layer. Because α is the ratio of resistances, it can be easily measured dynamically for qualitative insight into top/bottom differences. As χ is the ratio of resistivities, it requires detailed knowledge of the thickness and can be measured only at the end of each experiment. The χ measured for the dried film was χ­(*t* = ∞) = 106.7 ± 31.3, which confirms that the ratio of α is due to differences in resistivity, and thus carbon distribution, across the dried film thickness.

**Figure 5:**
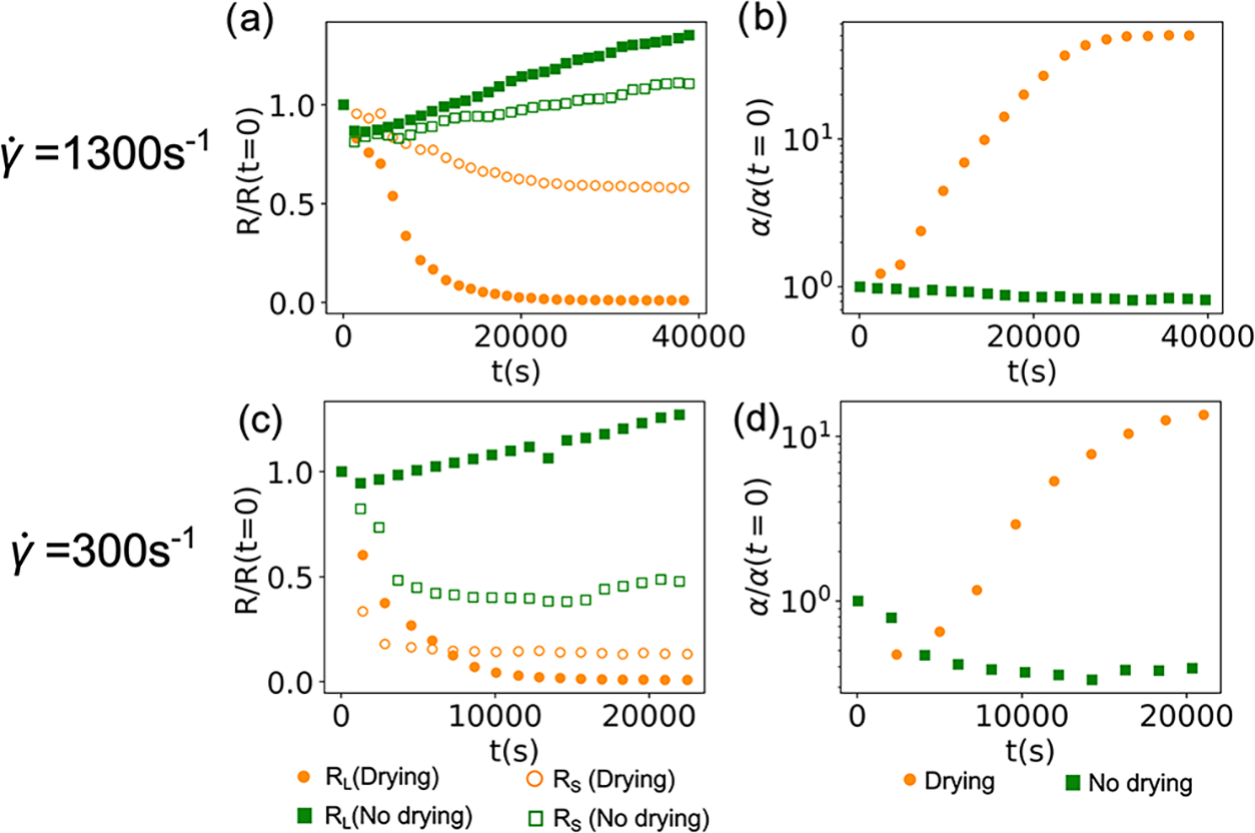
(a) Change in resistance measured by probes with *P* = 1 mm (filled) and *P* = 10 μm (unfilled) when an electrode slurry coated at γ̇ = 1300 s^–1^ is drying (orange circle) and when drying is suppressed (green square). (b) Change in normalized resistance ratio α corresponding to the change in resistance for (a) drying (orange circles) and no-drying (green squares) cases. Panels (c, d) are identical to panels (a, b) but for slurries coated at γ̇ = 300 s^–1^. The resistivity ratios immediately after shear are χ­(*t* = 0) = 2.0 ± 1.1 and 4.8 ± 5.1 for γ̇ = 1300 and 300 s^–1^, respectively.

There are two possible explanations for the increase in α with time. A concentrated layer of carbon particles may form at the top of a drying film. Our previous study showed that such evaporation-dominated drying causes α to increase with time.^19^ Alternatively, the shear imposed during coating may create a vertical gradient in the carbon concentration within the film. Nondrying experiments were conducted to separate the effects of drying and shear. The green squares in Figure 5(a) show the change in the resistance for both probes in the absence of drying. Initially, we observe a steep drop in resistance at early times after shear has stopped. This suggests that the high shear rate induces increased connections between the carbon particles. This finding is supported by data shown in Figure SI2, which shows that the elastic modulus increases with time after shear. At later times, the resistances measured by both sets of electrodes increase, which suggests that the connections between carbon black particles break with aging. α shows a near-horizontal line, suggesting the absence of gradients in the carbon microstructure from top to bottom over time. We can therefore conclude that a high shear rate during coating does not lead to vertical variations in carbon concentration. Thus, the drying dynamics must be responsible for the increase in α shown in Figure 5(a). This is a similar effect to that shown in modeling and experiments, whereby small particles are forced to the top of the drying film and larger particles are excluded to form a stratified film as a result of a concentrated layer of particles forming at the top.^22,23^ However, the osmotic driving forces in these studies may be different from those in this study due to the highly constrained mobility of the carbon aggregates. Another explanation could be the migration of carbon and binder through the emptying pores between active material particles via capillary action as reported by Westphal et al. and Jaiser et al.^11,12^

Next, we observe the impact of the shear rate on the electrode microstructure by repeating experiments at a much slower coating shear rate. Figure 5(c) shows the change in *R*_L_ (filled circles) and *R*_S_ (unfilled circles) for an electrode slurry coated at γ̇ = 300 s^–1^. Similar to the high-rate case, the resistance measured by both probes decreases as the film dries. However, in the low-rate case, *R*_L_ decreases faster than *R*_S_ such that α (Figure 5(d)) initially decreases below unity and then increases to over a factor of 10. Note that the χ measured for the dried film is much lower than the high-rate case with χ­(*t* = ∞) = 39.0 ± 15.4. The χ­(*t* = ∞) values are obtained as an average and standard deviation of three replicates. The replicates are depicted in Figure S1. The increase in α at large times is expected given the discussion of the high-rate case above. The interesting difference is the minimum that occurs at an early time, which cannot be explained solely by drying dynamics. For low-rate shear and no drying, shown in Figure 5(c,d) (green lines), we observe that *R*_L_ increases similar to the high-rate case, while *R*_S_ decreases significantly. This is most likely explained by sedimentation of the particles immediately after coating. Our previous model results showed that a decrease in α occurs due to sedimentation of the conductive material.^19^ However, this begs the question of why carbon settles at low rates but not at high rates.

Eberle et al. showed that the shear rate imposed on colloidal suspensions significantly affects the network microstructure of the colloidal gel.^24^ For example, larger and denser aggregates are formed after the cessation of flow from relatively low rates compared with highly sparse, more connected networks formed from relatively high rates. The relative magnitude of the shear rate is measured by comparison to the magnitude of interparticle forces, i.e., *M*′ = 6πμ_s_ *a* ^2^γ̇/*F*_max_. When *M*′ < 1, we expect that the aggregation of particles forms denser clusters and fewer network connections, while *M*′ > 1 leads to more network connections. Through combined rheological and small-angle neutron scattering experiments, Hipp et al. demonstrated that shearing a carbon black suspension below a critical shear rate of ∼500^–1^, defined quantitatively by the inverse Bingham number, causes the formation of large and dense agglomerates that induce sedimentation.^25^ Previously, we showed that the modulus for slurries initially sheared at 300 s^–1^ decreases significantly compared to the presheared state, while slurries sheared at 1300 s^–1^ showed an improved modulus compared to the presheared case.^1^ We qualitatively argued that this signifies that settling and poor network connections are formed for low-rate coatings. The no-drying results in Figure 5(a–d) quantitatively validate these arguments, whereby no structural changes were seen at high rates but significant changes are observed at low rates. Thus, the minimum observed in α for the low-rate case is explained by sedimentation of particles due to the loss of network connections.

In summary, microscale four-line probe measurements of resistivity suggest the following mechanism. High shear rate induces connections between carbon particles that decrease the resistivity and increase the colloidal stability. These connections decay very slowly, and evaporation dominates drying, such that the carbon forms a conductive layer at the electrode surface. Low shear rate, in contrast, does not form such connections, and carbon forms agglomerates. These agglomerates can sediment to form a bottom layer of low resistivity or migrate to form a top layer enriched in carbon. The competition between the sedimentation and evaporation processes causes a transient minimum in α.

While the results from the four-point probe are compelling, it is useful to measure the carbon distribution in the films directly. SEM and EDS experiments were completed to validate the model. Previously, SEM-EDS was used to measure carbon distribution in coated battery films.^1,26^ Figure 6 shows an example cross-sectional SEM image of an electrode coated at γ̇ = 1300 s^–1^ and the corresponding carbon map produced by EDS. Figure 7 shows the normalized concentration of carbon as a function of the normalized vertical position produced via an image analysis. Note that the curves represent an average over 1.05 mm of film length for each shear rate. These results show quantitatively that the top surface of the film (0.8 < *z*/*H* < 1) exhibits the highest carbon concentration for both shear rates, supporting the mechanisms discussed above. In the low-rate-coated film, the carbon concentration below *z*/*H* = 0.8 is substantially lower and remains relatively constant throughout the depth. In contrast, the high-rate-coated film shows a more gradual decrease in the carbon concentration with depth. The differences in the carbon distribution in the bottom of the films between high and low rate are subtle and cannot explain the differences observed in resistance, while the differences at the top of the film clearly indicate important parameters for modeling. Namely, the thickness of the concentrated layer at the top is used to parametrize the drying model. Although the carbon concentration at the top is lower, we expect the resistivity of the top layer of the high-rate film to be much lower than that of the low-rate film due to the better network connections achieved at high shear rates.

**Figure 6:**
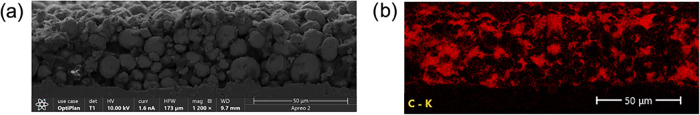
(a) Example SEM image for the cross section of a film coated at γ̇ = 1300 s^–1^. (b) Carbon map for the SEM image shown in panel (a) produced by EDS with carbon shown in red.

**Figure 7:**
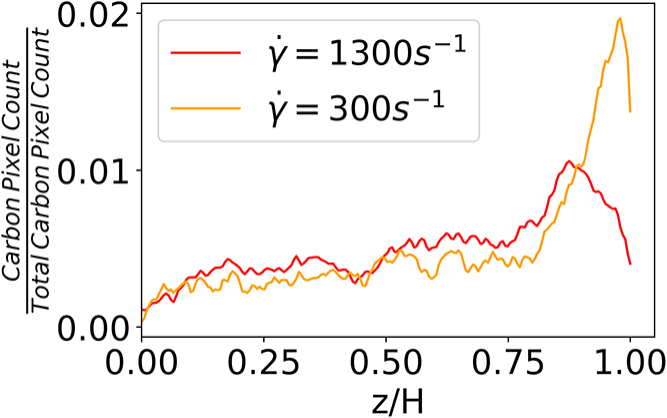
Plot showing the change in the number of red pixels depicting carbon concentration as a function of vertical position averaged over 6 images.

To verify our network connectivity hypothesis, we performed EFM on the final dried electrodes. Figure 8(a,b) shows EFM images of 1 mm regions of interest (ROIs) from dried electrodes coated at the two shear rates. Recall that the green pixel intensity reflects the local concentration of reduced AQ (the fluorophore). Black pixels correspond to areas where AQ remains unreduced, indicating limited electron conduction and, in turn, carbon connectivity. The colored bars below Figure 8(a,b) show the fractions of dark and bright pixels averaged over six images. The film coated at a low rate has twice the number of dark pixels compared to the high-rate film, indicating that coating at a high shear rate leads to better distribution of carbon and a stronger colloidal carbon network. While the EDS pixel integration in Figure 7 suggests that both films have a significant concentration of carbon at the top, the EFM analysis shows that there is a significant difference in carbon connectivity. These results are consistent with Narayan et al., who showed that high preshear rates yield small agglomerates in highly branched networks after shear cessation, whereas low preshear rates produce larger, fewer agglomerates that form networks with thicker, less numerous branches.^27^ Furthermore, the difference in carbon connectivities of the concentrated top layer has important implications for the model described below.

**Figure 8:**
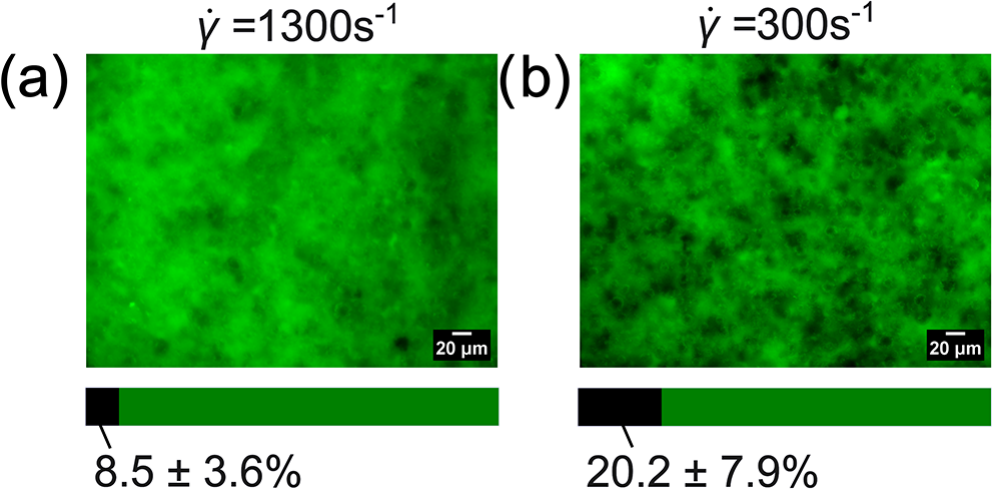
EFM images of the dried electrodes coated at (a) γ̇ = 1300 s^–1^ and (b) γ̇ = 300 s^–1^ with colored bars on the bottom depicting the ratio of dark to green areas as an average of 6 images.

The equations defined in Drying Model Development were solved to determine the drying mechanism in the film as a function of time. However, based on the experimental data, the low-rate and high-rate films have different drying physics. The high-rate film shows a concentrated top layer that grows as the film evaporates, while the low-rate film forms both a concentrated top layer and a sedimentation layer. The top layer forms after an initial time delay, while the sedimentation layer starts to form immediately. Additionally, the concentrated top layers in the high-rate and low-rate films differ. There is a much greater degree of carbon connectivity in the high-rate film. The evolutions of these modeled resistivity gradients for the high- and low-rate films are shown in Figure 9(a,b) respectively. The high-rate film is modeled via an evaporation model given by eq 2 coupled with a concentrating top layer thickness given by eq 5 and a change in resistivity of the top layer and bottom layer given by eqs 6 and 4, respectively. The low-rate film is modeled similarly with eqs 2,5,6 and 4 and coupled with the growth of a sedimentation layer given by eq 3 with a constant resistivity of ρ_b_. Note that the resistivity of the depleted slurry, neither the concentrated top layer nor the sedimentation layer, aka the blue layer in Figure 9(a,b), is given by eq 4. The parameters for these set of equations are given in Table 1.

**Table 1:** Model Parameters Used for Equations 1– [Disp-formula eq6] ^a^

γ̇ (s^–1^)	*t*_f_ (s)	*t*_sed_ (s)	*H*_dry_/*H*_0_	δ_t_/*H*_dry_	δ_m_/*H*_dry_	δ_b_/*H*_dry_	ρ_t_/ρ_0_	ρ_m_/ρ_0_	ρ_b_/ρ_0_	*A*
1300	44,300	0	0.5	0.25	N/A	0.75	0.0013	N/A	0.60	20*
300	21,000	2100	0.5	0.20	0.4	0.40	0.0016	0.25	0.25	11*

a All parameters were determined from experimental data except those marked with *, which were determined from a parameter estimation using α versus time data in Figure 5.

**Figure 9:**
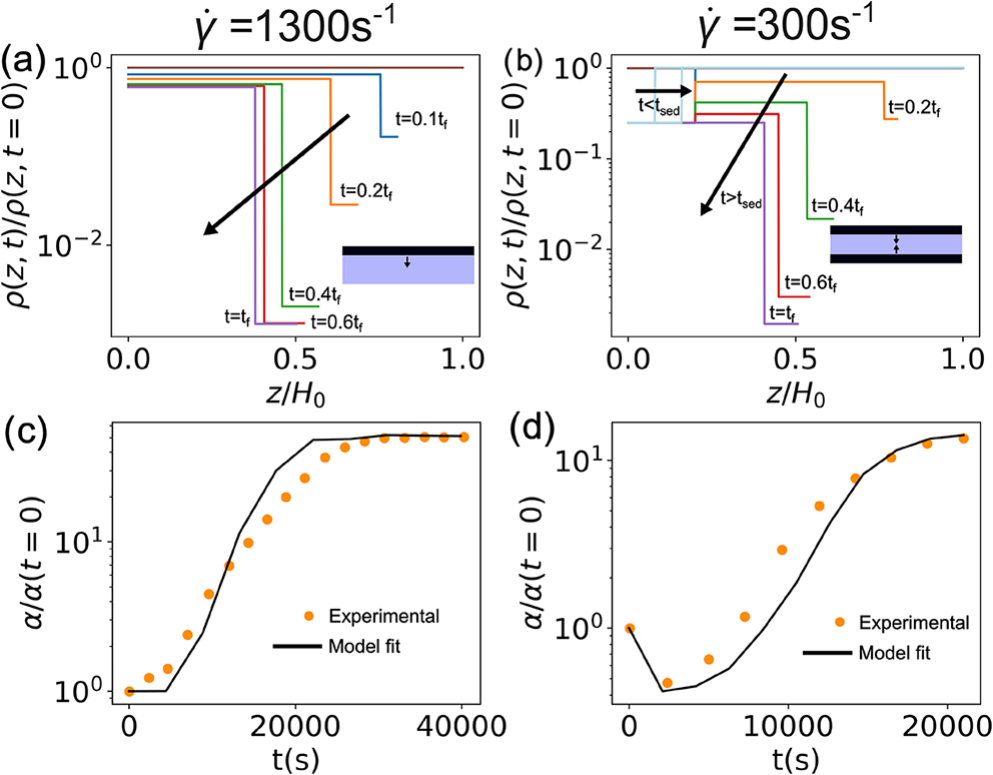
(a) Modeled vertical resistivity profiles at variable time for γ̇ = 1300 s^–1^. The black arrow indicates an increased time. The inset illustrates the drying mechanism. (b) Modeled vertical resistivity profiles at variable time for γ̇ = 300 s^–1^. The black arrow indicates increased time. The inset illustrates the drying mechanism. (c) Change in α measured as an electrode slurry coated at γ̇ = 1300 s^–1^ dries and the corresponding model fit. (d) Change in α measured as an electrode slurry coated at γ̇ = 300 s^–1^ dries and the corresponding model fit.

Equations 1– [Disp-formula eq6] require a total of ten parameters to simulate the change in resistance for a given drying experiment. However, nine of these ten parameters can be approximated experimentally using the data discussed above. For example, *H*_dry_/*H*_0_ is the dry:wet thickness ratio measured experimentally to be 0.5. The time for sedimentation, *t*_sed_, and total drying time, *t*_f_, are determined from Figure 5 and are denoted by the time required to reach steady state for the nondrying film and drying film, respectively. Recall that *t*_sed_ = 0 for γ̇ = 1300 s^–1^ because no sedimentation occurs. δ_t_/*H*_dry_ is determined from the carbon peak position and width in the SEM study in Figure 7. Estimations of δ_b_/*H*_dry_ and δ_m_/*H*_dry_ are different for the two shear rates. For the high-rate film, δ_b_/*H*_dry_ = 1 – δ_t_/*H*_dry_ and δ_m_/*H*_dry_ = 0 because there are only two layers. For the low-rate film, δ_b_/*H*_dry_ = 0.4, which is the upper limit that the smaller-spaced probe can detect,^19^ and the middle layer thickness is simply calculated using δ_m_/*H*_dry_ = 1 – δ_t_/*H*_dry_ – δ_b_/*H*_dry_. ρ_b_/ρ_0_ is taken to be the asymptotic value of *R*/*R*(*t* = 0) for the small spaced probes in Figure 5(a,c), since *R*/*R*(*t* = 0) is equivalent to ρ_b_/ρ_0_ for the small spaced probes.^19^ Since the carbon concentration of the middle layer for the low-rate film is similar to that of the bottom, see Figure 7, we assume that the two layers are microstructurally similar such that ρ_m_/ρ_0_ = ρ_b_/ρ_0_. ρ_t_/ρ_0_ is determined by assuming the layers are resistors in parallel, which gives

7 $$\frac{1}{R_{L}}=\sum_{i=1}^{N}\frac{1}{R_{i}}$$

8 $$\rho_{i}=\frac{R_{i}\pi \delta_{i}}{\ln\left(2\right)}$$

where *R*_*i*_ is the effective resistance of layer *i* of the *N*-layer film and δ_*i*_ is the final thickness of layer *i*.^28^ After these nine parameters were restricted to experimentally measured values or approximations, the only remaining fitting parameter is *A*, which governs the rate at which the concentration of the top layer increases. Unfortunately, there exists no measured quantity to quantify this parameter. Thus, the magnitude of *A* was determined by visual comparison of calculated α versus time with experimental trends in Figure 9(c,d). The effect of varying *A* is shown in Figure S3.

Two important conclusions can be drawn from these model results. First, that the increase in α observed experimentally at both shear rates can only be caused by the formation of a concentrated top layer that is much less resistive than the bottom layer. Second, the minimum in α observed in the low shear case at early times can be explained by the formation of a concentrated sediment layer at the bottom. The qualitative agreement between the model and experiment with only one fitting parameter validates the proposed drying mechanisms.

In a previous work by our group, electrodes with the same formulation demonstrated superior electrochemical performance when coated at γ̇ = 1300 versus 300 s^–1^ when dried at room temperature and not calendered.^1^ Note that calendering removes the shear and drying history of a sample as shown by Pan et al.^26^ Since carbon serves as the conductive additive, a higher carbon concentration near the top surface appears to enhance the battery performance. This observation contrasts with findings by Yari et al., who reported improved performance when carbon concentration was higher near the current collector in their two-layered cathodes made with a similar formulation.^8^ The disagreement may be explained by the work of Saraka et al., who suggest that the relative distribution of carbon and NMC particles—rather than the absolute vertical distribution of carbon alone—is more critical to the overall electrode performance.^1^ An interesting extension of this work would be to study the effect of temperature on the final distribution of carbon since there is some disagreement in literature on whether a high evaporation rate would minimize^1^ or exacerbate^11,12^ the heterogeneity. This would also shed light on the reasons for improved electrode performance when drying at high temperatures.^1^

## Conclusion

This study demonstrates an in situ microscale resistivity measurement technique to investigate the drying behavior of LIB electrode films as a function of the coating shear rate. Resistivity measurements indicate that low shear rates (γ̇ = 300 s^–1^) promote the formation of larger carbon aggregates that tend to sediment, whereas higher shear rates (γ̇ = 1300 s^–1^) help maintain a more uniform particle distribution. The final resistivity ratio, χ­(*t* = ∞), reveals that both films exhibit higher resistivity at the bottom than at the top with a more pronounced gradient in low-coating-rate films. This resistivity gradient is caused by the growth of a consolidation front during drying, which drives carbon particle migration to the top of the film. In low-rate films, larger carbon aggregates both sediment and migrate, resulting in a less uniform carbon distribution.

Quantitative analysis of carbon EDS maps confirmed a higher concentration of carbon black at the top surface for films coated at both shear rates, supporting the proposed carbon migration mechanism. However, when normalized by the total carbon content, low-rate films exhibited a greater relative abundance of carbon particles near the surface—contrary to what the χ­(*t* = ∞) values suggest. This implies that the carbon network in low-rate films is less interconnected, allowing for individual particles to migrate more easily. EFM measurements corroborate this interpretation, revealing reduced network connectivity near the surface for low-rate coatings. The proposed mechanisms were then modeled by considering the above mechanisms in microstructural evolution. The quantitative agreement between the experimental and model trends in α serves as a validation of the proposed microstructural evolution. Overall, these results offer a comprehensive understanding of how the shear rate influences the microstructural development of LIB cathodes during drying and its implications for electrode performance using a low-cost, in situ method.

## Supplementary Material



## Notes

Replicates of drying experiments, rheology showing the change in elastic modulus of the electrode slurry after preshear, and model results with variable parameter *A* (PDF)

## Letters/Symbols

*A*: constant for the rate of top layer concentration, N/A
*H*(*t*): transient film thickness, mm
*H*_0_: initial film thickness, mm
*H*_dry_: dry film thickness, mm
*P*: spacing between adjacent electrodes, mm
*R*: resistance, Ω
*R*_L_: resistance observed by large spaced probe, Ω
*R*_S_: resistance observed by small spaced probe, Ω
*t*: time, s
*t*_f_: final drying time, s
*t*_sed_: sedimentation time, s
*U*: sedimentation velocity, mm/s
*x*: 3D horizontal spatial coordinate, mm
*z*: 3D vertical spatial coordinate, mm
α: *R*_S_/*R*_L_, N/A
δ_b_: final bottom layer thickness, mm
δ_b_(*t*): transient bottom layer thickness, mm
δ_t_: final top layer thickness, mm
δ_t_(*t*): transient top layer thickness, mm
ρ: resistivity of the film, Ωmm
ρ_0_: initial resistivity of the film, Ωmm
ρ­(*t*): transient resistivity of the film, Ωmm
ρ­(*z*, *t*): resistivity of the film as a function of the vertical coordinate and time, Ωmm
ρ_b_: final resistivity of the bottom layer, Ωmm
ρ_b_(*t*): transient resistivity of the bottom layer, Ωmm
ρ_t_: final resistivity of the top layer, Ωmm
ρ_t_(*t*): transient resistivity of the top layer, Ωmm
ρ_m_: final resistivity of the middle layer, Ωmm
ρ_m_(*t*): transient resistivity of the middle layer, Ωmm
ρ_L_: resistivity observed by large spaced probe, Ωmm
ρ_S_: resistivity observed by small spaced probe, Ωmm
ρ_sed_: resistivity of the sediment layer, Ωmm
τ_E_: evaporation time constant, s
ϕ: electric potential, V
Φ: particle volume fraction, N/A
χ: ρ_S_/ρ_L_, N/A

## References

[1] Saraka R. M., Morelly S. L., Tang M. H., Alvarez N. J. (2020). Correlating Processing
Conditions to Short- And Long-Range Order in Coating and Drying Lithium-Ion
Batteries. ACS Appl. Energy Mater..

[2] Morelly S. L., Alvarez N. J., Tang M. H. (2018). Short-range
contacts govern the performance
of industry-relevant battery cathodes. J. Power
Sources.

[3] Peterson S. W., Wheeler D. R. (2014). Direct Measurements of Effective Electronic Transport
in Porous Li-Ion Electrodes. J. Electrochem.
Soc..

[4] Dominko R., Gaberšček M., Drofenik J., Bele M., Jamnik J. (2003). Influence of carbon black distribution on performance
of oxide cathodes for Li ion batteries. Electrochim.
Acta.

[5] Dominko R., Gaberscek M., Drofenik J., Bele M., Pejovnik S., Jamnik J. (2003). The role of carbon black distribution in cathodes for
Li ion batteries. J. Power Sources.

[6] Bauer W., Nötzel D., Wenzel V., Nirschl H. (2015). Influence of dry mixing
and distribution of conductive additives in cathodes for lithium ion
batteries. J. Power Sources.

[7] Entwistle J., Ge R., Pardikar K., Smith R., Cumming D. (2022). Carbon binder domain
networks and electrical conductivity in lithium-ion battery electrodes:
A critical review. Renewable Sustainable Energy
Rev..

[8] Yari S., Hamed H., D’Haen J., Bael M. K. V., Renner F. U., Hardy A., Safari M. (2020). Constructive
versus Destructive Heterogeneity
in Porous Electrodes of Lithium-Ion Batteries. ACS Appl. Energy Mater..

[9] Gao X., Xu J. (2024). Carbon Binder
Domain Inhomogeneity in Silicon-Monoxide/Graphite Composite
Anode by 2D Multiphysics Modeling. Adv. Sci..

[10] Ramasubramanian B., Sundarrajan S., Chellappan V., Reddy M. V., Ramakrishna S., Zaghib K. (2022). Recent Development
in Carbon-LiFePO4 Cathodes for Lithium-Ion
Batteries: A Mini Review. Batteries.

[11] Westphal B. G., Kwade A. (2018). Critical electrode
properties and drying conditions causing component
segregation in graphitic anodes for lithium-ion batteries. J. Energy Storage.

[12] Jaiser S., Müller M., Baunach M., Bauer W., Scharfer P., Schabel W. (2016). Investigation of film solidification
and binder migration
during drying of Li-Ion battery anodes. J. Power
Sources.

[13] Gören A., Cíntora-Juárez D., Martins P., Ferdov S., Silva M. M., Tirado J. L., Costa C. M., Lanceros-Méndez S. (2016). Influence
of Solvent Evaporation Rate in the Preparation of Carbon-Coated Lithium
Iron Phosphate Cathode Films on Battery Performance. Energy Technol..

[14] Li C.-C., Wang Y.-W. (2011). Binder Distributions in Water-Based and Organic-Based
LiCoO2 Electrode Sheets and Their Effects on Cell Performance. J. Electrochem. Soc..

[15] Chang W. J., Lee G. H., Cheon Y. J., Kim J. T., Lee S. I., Kim J., Kim M., Park W. I., Lee Y. J. (2019). Direct Observation
of Carboxymethyl Cellulose and Styrene-Butadiene Rubber Binder Distribution
in Practical Graphite Anodes for Li-Ion Batteries. ACS Appl. Mater. Interfaces.

[16] Jaiser S., Kumberg J., Klaver J., Urai J. L., Schabel W., Schmatz J., Scharfer P. (2017). Microstructure
formation of lithium-ion
battery electrodes during drying – An ex-situ study using cryogenic
broad ion beam slope-cutting and scanning electron microscopy (Cryo-BIB-SEM). J. Power Sources.

[17] Lim S., Ahn K. H., Yamamura M. (2013). Latex migration
in battery slurries
during drying. Langmuir.

[18] Kumberg J., Baunach M., Eser J. C., Altvater A., Scharfer P., Schabel W. (2021). Influence of Layer
Thickness on the Drying of Lithium-Ion
Battery Electrodes—Simulation and Experimental Validation. Energy Technol..

[19] Baburoglu E., Tang M. H., Alvarez N. J. (2025). Microscale
Electrical Resistivity
Measurements to Investigate Particle Distribution. Langmuir.

[20] Negrete K., Tang M. H. (2024). Visualizing and Quantifying Electronic
Accessibility
in Composite Battery Electrodes using Electrochemical Fluorescent
Microscopy. J. Electrochem. Soc..

[21] Cardinal C. M., Jung Y. D., Ahn K. H., Francis L. F. (2010). Drying regime maps
for particulate coatings. AIChE J..

[22] Fortini A., Martín-Fabiani I., Haye J. L. D. L., Dugas P. Y., Lansalot M., D’Agosto F., Bourgeat-Lami E., Keddie J. L., Sear R. P. (2016). Dynamic Stratification
in Drying Films of Colloidal Mixtures. Phys.
Rev. Lett..

[23] Cusola O., Kivistö S., Vierros S., Batys P., Ago M., Tardy B. L., Greca L. G., Roncero M. B., Sammalkorpi M., Rojas O. J. (2018). Particulate Coatings via Evaporation-Induced Self-Assembly
of Polydisperse Colloidal Lignin on Solid Interfaces. Langmuir.

[24] Eberle A. P. R., Martys N., Porcar L., Kline S. R., George W. L., Kim J. M., Butler P. D., Wagner N. J. (2014). Shear viscosity
and structural scalings in model adhesive hard-sphere gels. Phys. Rev. E.

[25] Hipp J. B., Richards J. J., Wagner N. J. (2019). Structure-property
relationships
of sheared carbon black suspensions determined by simultaneous rheological
and neutron scattering measurements. J. Rheol..

[26] Pan, S. Effects of Binder and Solvent on Cathode Manufacturing for Li-Ion Batteries. Ph.D. thesis, Drexel University, 2024.

[27] Narayanan A., Mugele F., Duits M. H. (2017). Mechanical History Dependence in
Carbon Black Suspensions for Flow Batteries: A Rheo-Impedance Study. Langmuir.

[28] Chen Y. Y., Juang J. Y. (2016). Finite element analysis and equivalent
parallel-resistance
model for conductive multilayer thin films. Meas. Sci. Technol..

