# Supplementary material for: Measuring Dynamic Gradients in Drying Battery Electrode Coatings via Microscale Resistivity
Source: Langmuir. 2025 Oct 28;41(44):29976–84. doi: 10.1021/acs.langmuir.5c04644 (PMC12613802; doi:10.1021/acs.langmuir.5c04644)
Supplement: Supplementary file 1 [file la5c04644_si_001.pdf]

Measuring dynamic gradients in drying battery electrode coatings via microscale resistivity: Supporting Information

Emre Baburoglu[1], Karla Negrete[2], Maureen H. Tang[3], Nicolas J. Alvarez[3*]

[1*]Materials Science and Engineering, Drexel University, 3141 Chestnut Street, Philadelphia, 19104, PA, USA.
[2*]Mechanical Engineering and Mechanics, Drexel University, 3141 Chestnut Street, Philadelphia, 19104, PA, USA.
[3*]Chemical and Biological Engineering, Drexel University, 3141 Chestnut Street, Philadelphia, 19104, PA, USA.

*Corresponding author(s). E-mail(s): alvarez@drexel.edu;
Contributing authors: eb937@drexel.edu; kn583@drexel.edu; mhtang@drexel.edu;

Number of pages: 3
Number of figures: 2
Number of tables: 0

Contents

1 Resistance Measurement Replicates **S2**

2 Rheological Measurements **S3**

3 Fitting parameter A **S4**

1 Resistance Measurement Replicates

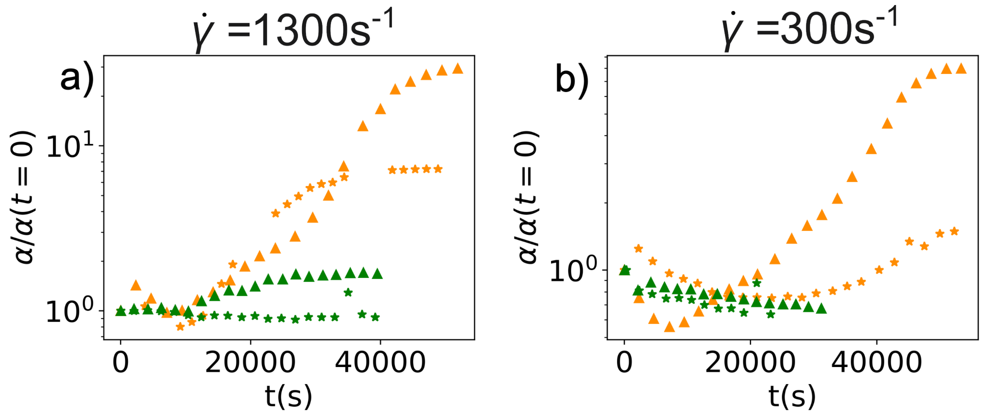

Figure S 1 Replicates for four-line measurements shown in Figure 5. a) Two replicates of the change in α (marked as stars and squares) when an electrode slurry coated at $\dot{\gamma}$=1300 s^{-1} is drying (orange) and when drying is suppressed (green). b) is identical to a) but for slurries coated at $\dot{\gamma}$=300 s^{-1}.

Figure S1(a) shows repeat experiments for the measured change in α when an electrode slurry is coated at $\dot{\gamma}$=1300 s^{-1} shown in Figure 5(b). Similar to Figure 5(b), when drying is allowed (orange markers), α increases. Dissimilar to Figure 5(b), there are small fluctuations observed at early times for both replicates. These may be explained by the competition between the decrease in resistance caused by an increase in carbon concentration and the increase in resistance caused by the decreased in thickness at early times. More likely, they are caused by data noise. The irregular gap between data points seen in the replicate with star markers is due to corrupted data that was omitted. When drying is suppressed (green markers), similar to Figure 5(b), α is approximately horizontal in one replicate (stars) but increases slightly in the other. This increase in α could be due to shear-banding causing the bottom of the film to experience a different shear rate than the top, increasing it's resistivity.

Figure S1(b) shows repeat experiments for the measured change in α when an electrode slurry is coated at $\dot{\gamma}$=300 s^{-1} shown in Figure 5(d). Similar to Figure 5(b), when drying is allowed (orange markers), both replicates exhibit a minimum in α. The variation of the timescale for the occurrance of this minimum is likely due a combination of the variation in drying rate and extent of sedimentation between experiments. The slight increase in α observed in the replicate with star markers at early times is likely due to the same reason as the fluctuations observed in the high-rate case. When drying is suppressed, similar to Figure 5(b),α decreases. The variation in the extent of this decrease is likely due to the variation in the extent of sedimentation.

Figure S1(a) shows repeat experiments for the measured change in α when an electrode slurry is coated at $\dot{\gamma}$ = 1300 s^{-1}, as shown in Figure 5(b). Similar to Figure 5(b), when drying is allowed (orange markers), α increases. Unlike Figure 5(b), small

fluctuations are observed at early times in both replicates. These fluctuations may be caused by the competing effects of decreasing resistance due to increasing carbon concentration and increasing resistance due to reduced thickness at early times. More likely, however, they result from data noise. The irregular gaps between data points in the replicate with star markers are due to corrupted data that were omitted. When drying is suppressed (green markers), α is approximately constant in one replicate (stars) but increases slightly in the other. This increase in α could be due to shear banding, which may cause the bottom of the film to experience a different shear rate than the top, thereby increasing its resistivity.

Figure S1(b) shows the corresponding repeat experiments at $\dot{\gamma} = 300$ s^{-1}, as in Figure 5(d). Similar to Figure 5(b), when drying is allowed (orange markers), both replicates exhibit a minimum in α. The variation in the timescale at which this minimum occurs is likely due to differences in drying rate and the extent of sedimentation between experiments. The early-time increase in α seen in the star-marked replicate is likely caused by the same factors as the fluctuations in the high-rate case. When drying is suppressed (green markers), α decreases, as in Figure 5(b). The variation in the extent of this decrease is likely due to differences in the degree of sedimentation.

2 Rheological Measurements

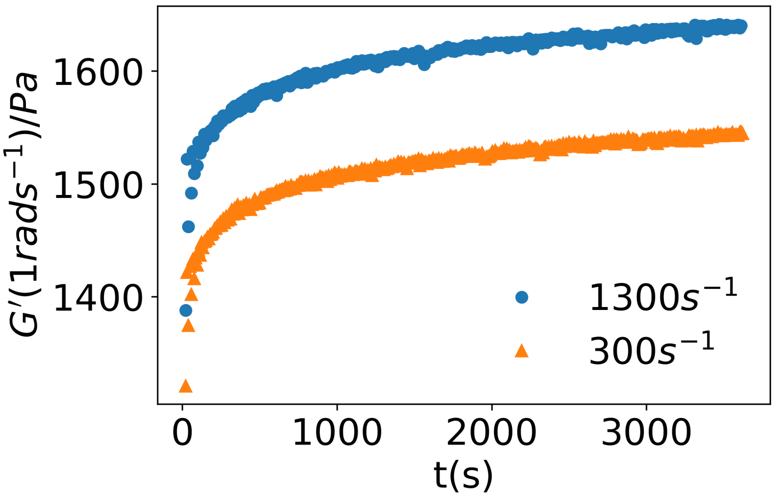

Figure S 2 Change elastic modulus after pre-shear at $\dot{\gamma}$=1300 s^{-1}(circles) and 300 s^{-1}(triangles) measured at a frequency of of 1 rad s^{-1} and an amplitude of 1%.

Figure S2 shows the change in the elastic modulus (G') of the battery slurry after being sheared at $\dot{\gamma}$=1300 s^{-1} and 300 s^{-1} for 1 and 3 seconds respectively. This measurement is done using a Discovery HR3 rotational rheometer by TA instruments at a frequency of 1 rad s^{-1} and an amplitude of 1%. G' increases after shear for both

shear rates which indicates network formation. This explains the drop in resistance measured by the larger-spaced probes at early times seen in Figure 5(a).

3 Fitting parameter A

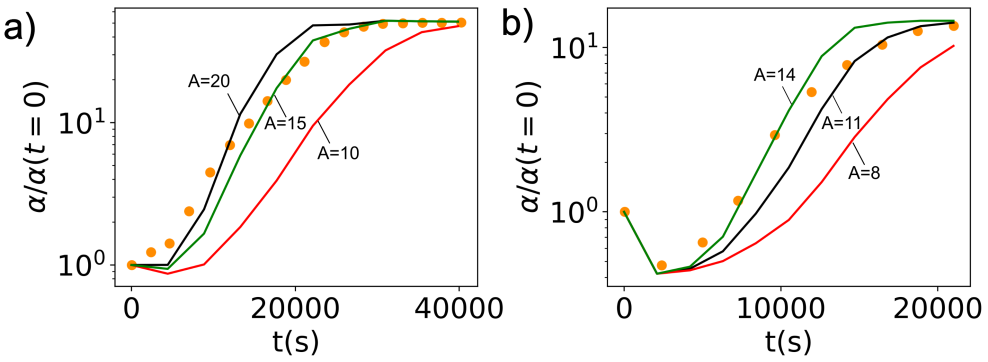

Figure S 3 Impact of changing A on the model fit for a) the high and b) low rate cases. Orange circles are the experimental data and the lines are model fits for different values of A.

Figure S3 shows the impact of changing the fitting parameter, A, which governs the rate at which the concentration of the top layer increases during drying. For both the low and the high rate case, increasing A increases the rate of increase in α. Since it is part of an exponential function (Eq **??**) when A is too high, α plateaus too early. When A is too low, α either does not reach the $\alpha(t=\infty)$ value (Figure S3(b)) or exhibits a small decrease at early times. This decrease is due to the competing effects of the shrinkage of film thickness and the increase in the concentration of the top layer on R_L. The decrease in thickness increases R_L, while increase in the concentration of the top layer decreases it. If the thickness shrinks faster than the top layer concentrates, α decreases slightly.
